# Co-delivery of siRNA and cisplatin via electrospun Nanofibrous membranes for synergistic treatment of malignant melanoma

**DOI:** 10.1016/j.heliyon.2024.e37517

**Published:** 2024-09-06

**Authors:** Xuewei Zhang, Guoxing Zheng, Zibin Zhou, Mingyu Zhu, Shijie Tang

**Affiliations:** a Department of Plastic Surgery and Burn Center, Second Affiliated Hospital, Shantou University Medical College, Shantou, Guangdong, 515000, China; b Department of Spine Surgery, Second Affiliated Hospital, Shantou University Medical College, Shantou, Guangdong, 515000, China; c Division of Pharmaceutical Chemistry and Technology, Faculty of Pharmacy, University of Helsinki, Helsinki, 00014, Finland; d Department of Materials Science and Engineering, Southern University of Science and Technology, Shenzhen, Guangdong, 518055, China

**Keywords:** Malignant melanoma, Drug delivery, Ferroptosis, Nano fiber, Gene therapy

## Abstract

Tumor recurrence and metastasis remain formidable challenges in clinical oncology. Although surgery is an effective treatment for early-stage solid tumors, residual cancer cells can lead to subsequent recurrence or metastasis. Conventional treatments for melanoma, such as anti-tumor medications and gene therapy, have distinct limitations. The rapid systemic distribution of anti-tumor drugs poses a significant challenge, often resulting in notable side effects and inadequate drug concentrations at the tumor site. Melanoma (MM), a deadly form of skin cancer, is known for its high mortality rate. In this study, we propose a novel strategy for treating MM by combining the controlled release of chemotherapeutic drugs encapsulated within Metal-Organic Frameworks (MOFs) and liposomes with gene therapy targeting Minichromosome Maintenance Proteins 4 (MCM4) using electrospinning and surface modification techniques. *In vitro* and *in vivo* results confirmed that this hierarchical membrane system can effectively deliver therapeutic MCM4 siRNA and release cisplatin to inhibit tumor growth. Furthermore, we demonstrated that MCM4 silencing promoted the sensitivity of melanoma cells to ferroptosis both *in vitro* and *in vivo*. The proposed strategy, by allowing for a controlled and sustained release of medication, could alleviate the challenges in drug delivery and aid in prevent tumor recurrence.

## 1 Introduction

Preventing tumor recurrence and metastasis remains a significant challenge in clinical cancer treatment. Currently, surgical resection is the most direct and effective approach for treating early-stage solid tumors. However, residual tumor cells and tissues can cause recurrence or metastasis months or even years after surgery [1,2]. Consequently, various chemotherapy drugs and bioactive compounds are employed as adjuvant treatments following tumor resection [[3], [4], [5]]. Despite their efficacy, many therapeutic agents, such as quercetin [6], citric acid [7], and tamoxifen [8], face limitations including poor water solubility, lack of specificity, and potential harm to normal tissues [9]. Gene therapy has gained prominence as a targeted preventive or therapeutic strategy [10]. By manipulating gene transcription and translation processes, gene therapy can suppress or restore the function of specific genes, thereby attenuating the malignant features of cancer cells [11].

A critical aspect of gene therapy delivery involves the meticulous selection of an appropriate gene delivery vector [12,13]. This vector must specifically target tumor cells while not compromising the healthy immune system or causing toxic reactions [14,15]. Additionally, it is crucial for the integrated genes to retain their functionality and be shielded from neighboring enzymes, ensuring they can execute their desired therapeutic actions while successfully maneuvering through complex obstacles within the tissues. A novel perspective on cancer treatment focuses on enhancing cell sensitivity to ferroptosis to induce cell death [16], which has shown significant implications in malignant melanoma [17]. Minichromosome Maintenance Proteins 4 (MCM4) [18], a key protein involved in initiating DNA replication, has garnered increasing attention due to its connection with important ferroptosis-related genes [19], such as Glutathione Peroxidase 4 (GPX4) and Tumor Protein p53 (TP53) [20]. Decreasing GPX4 levels result in the buildup of phospholipid peroxides, a crucial step in triggering ferroptosis [21]. Therefore, MCM4 has been proposed as a novel ferroptosis target in melanoma treatment.

Electrospun membranes have demonstrated significant advantages as a delivery system in cancer research [22,23]. These membranes leverage their inherent properties to deliver anti-tumor drugs and genes simultaneously, combining various therapeutic modalities for comprehensive tumor therapy [24]. Recent advancements in drug delivery systems aim to improve therapeutic efficacy and provide targeted treatment in cancer therapy. For instance, the use of bioactive glass fiber dressings in combination with chemotherapy has shown promise in post-surgical melanoma treatment [25] and similar strategies have been explored to enhance chemotherapy for pancreatic cancer [26]. Additionally, the design of targeted drug delivery systems that can simultaneously address multiple facets of tumor treatment. For example, alendronate-triggered dual-stage prodrug nanoparticles are engineered to enhance tumor penetration and activate the STING pathway in osteosarcoma [27]. Synthetic polymers, renowned for their excellent biocompatibility and biodegradability [28], have extensive applications in the biomedical field. Among these polymers, polylactic acid (PLA) stands out as a preferred choice for electrospinning applications due to its superior elasticity and mechanical strength [29,30]. To achieve stable and sustained drug release at tumor sites, research efforts have been made to modify the basic fiber scaffold architecture to enhance stability and pharmacokinetics [31,32]. Metal-organic frameworks (MOFs), characterized by their porous structure, have emerged as effective drug carriers capable of adsorbing and storing drugs effectively [33]. The porous nature of MOFs facilitates the gradual and controlled release of drugs over a predetermined period, rendering them pivotal components in targeted drug delivery systems [34].

**Fig. 1:**
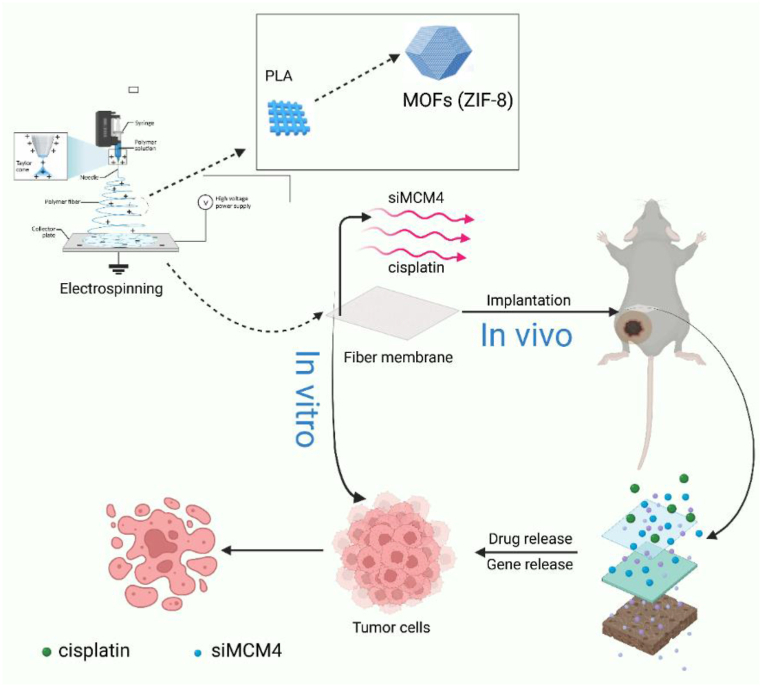
Schematic illustration of the use of PLA@MOF for melanoma therapy.

In this work, we developed a dual-release system using liposomes for MCM4-siRNA encapsulation and MOFs for chemotherapeutic drug delivery (Fig. 1). The anti-tumor efficacy of the co-delivery system was evaluated in both melanoma cells and a mouse model of melanoma. We demonstrated the effect of MCM4 silencing on the sensitivity of melanoma cells to ferroptosis both *in vitro* and *in vivo*. By demonstrating significant anti-tumor activity and biosafety of the delivery system, our study offers a viable platform for delivering multiple therapeutic agents to achieve multi-modal anticancer therapy.

## 2 Materials and methods

### 2.1 Materials

Polylactic acid (PLA, Mw = 30000, Shenzhen Weike Experimental Technology Co., Ltd., China), Cisplatin (Beyotime, Shanghai, China), Erastin (Ferroptosis Inducer), Cell Counting Kit-8 (CCK-8), Malondialdehyde (MDA) Assay Kit, reduced glutathione and oxidized glutathione disulfide (GSSG) Assay Kit, 2′,7′-dichlorodihydrofluorescein diacetate (DCFH-DA), Calcein-AM/PI double stain kit (Beyotime, Shanghai, China), ELISA kits for IL-1β, IL-6, and TGF-β, and assay kits for ALT (Alanine Aminotransferase), ALP (Alkaline Phosphatase) and AST (Aspartate Aminotransferase) (Zeye Biotech, Shanghai, China), MCM4 siRNA (siMCM4), and control siRNA (siNC, Gene Pharma Co., Ltd., Suzhou, China) were used in this study.

### 2.2 Sample preparation and characterization

PLA powder (200 mg) was dissolved in 5 mL of acetone. This mixture was gradually added to a stirred conical flask, and the water bath temperature was maintained at 60 °C. The solution was continuously stirred for 4 h to ensure complete dissolution. Then, the solution was transferred into a 20 mL syringe, which was mounted onto the injection pump of an electrospinning machine. A stainless steel needle and an appropriate air-assisted electrospinning nozzle were attached, with the needle connected to the positive electrode of a high-voltage power supply (set at 8 kV) and the receiving roller linked to the negative electrode (at −2 kV).

To fabricate a dual-delivery membrane, 100 μL of cisplatin was added to 5 mL of acetone. After ultrasonic dispersion, the mixture underwent adsorption on a shaking table for 4 h to allow for cisplatin loading onto the ZIF-8 nanoparticles. The drug-loaded ZIF-8 was then incorporated into the PLA solution and electrospun into a fibrous membrane. The content of MOF has a significant effect on the spun fiber, the high content of MOF will produce obvious precipitation. In our selection of a MOF concentration of 10 %, there was no significant precipitation and no significant change in the morphology of the nanofibers (Fig. S1). The PLA membrane was immersed in 100 mL of a 10 mmol L^−1^ Tris solution, to which 200 mg of dopamine (DA) monomer was added. The polymerization of the dopamine coating proceeded at room temperature for 8 h, after which the dopamine-modified PLA membrane was placed into a liposome solution containing siRNA and maintained at 4 °C for 30 min to facilitate siRNA loading. The gene-loaded PLA membrane was then freeze-dried for long-term storage.

Therefore, the samples were divided into five groups: (1) blank PLA membrane (NF), (2) PLA membrane encapsulating MOF (MOF-NF), (3) Cisplatin-loaded MOF group (D@MOF-NF), (4) MCM4 siRNA-modified group (G@NF), and (5) co-delivery of cisplatin and siRNA group (G-D@MOF-NF). The electrospun films (with a diameter of 10 mm) were coated with gold. Scanning electron microscopy (SEM, TESCAN) and energy dispersive spectroscopy (EDS) analyses were performed at 10 kV for sample morphology characterization and elemental mapping, respectively.

### 2.3 Cisplatin release test

G-D@MOF-NF membrane was immersed in 3 mL PBS (pH = 7.4 and pH = 5.2) at 37 °C with shaking. At predetermined time points, the solution was collected and added an equal volume of fresh buffer solution. The drug release amount was calculated using an UV spectrophotometer.

### 2.4 mRNA on G-D@MOF-NF membrane characterization and release

To confirm that the mRNA is successfully assembled on the surface of the G-D@MOF-NF membrane, the FAM-labeled mRNA on G-D@MOF-NF membrane was observed using a fluorescence confocal microscopy. The gene release test is performed under the same conditions as cisplatin release. Quantify the mRNA release using a plate reader (M200 PRO, Austria) at the absorption wavelength of 450 nm.

### 2.5 Cisplatin uptake test

The cellular uptake of cisplatin was measured according to literature reported method [35]. Briefly, Melanoma cell lines B16F0 and A375 are seeded at 1 × 10^6^ cells per well in a 6-well plate and cultured at 37 °C with 5 % CO_2_ for 12 h. Then, the G-D@MOF-NF membrane was added and co-incubated with melanoma cells for 0.5, 2, and 6 h. At each timepoint, the cells were washed with PBS, digest the cells with aqua regia, and analyze the platinum content using ICP-MS (PE Avio 200, China).

### 2.6 Cell lines and transfection

Melanoma cell lines B16F0 and A375 (Shanghai Qincheng Biotechnology Co., Ltd., China) were cultured at 37 °C with 5 % CO_2_ in Dulbecco's Modified Eagle's Medium (DMEM) supplemented with 10 % fetal bovine serum (FBS, Gibco, USA) and 1 % penicillin‒streptomycin. Cells were seeded in 6-well plates at a density of 1.0 × 10^5^ cells per well one day prior to transfection, and approximately 50 % confluence was achieved at the time of transfection. For each transfection sample, the following steps were performed. First, the siRNA stock solution was diluted to a concentration of 1 μM. Then, 4 μL of this diluted siRNA solution was added to 200 μL of Opti-MEM® Reduced Serum Medium and briefly mixed. This mixture was allowed to rest at room temperature for 5 min. In a separate sterile 1.5 mL centrifuge tube, 6 μL of siRNA-Mate transfection reagent was combined with 200 μL of Opti-MEM and incubated for 5 min. Subsequently, the siRNA and transfection reagent solutions were mixed and incubated for 20 min at room temperature to allow the formation of siRNA-lipid complexes. Finally, the siRNA-lipid complex mixture was gently added to each well containing cells and culture medium. The plates were gently rocked to ensure uniform distribution and then incubated in a CO_2_ incubator at 37 °C for 48 h. The siRNA target sequences used in this study are provided in the supplementary information: Table S1.

### 2.7 Co-expression analysis of ferroptosis-related genes and MCM4

The co-expression relationships between key genes related to ferroptosis and MCM4 were analyzed using the Gene Expression Profiling Interactive Analysis (GEPIA) database, which is based on The Cancer Genome Atlas (TCGA) and Genotype-Tissue Expression (GTEx) datasets [36]. The GEPIA database is a valuable resource for determining gene expression patterns, providing important insights into the co-expression pattern of MCM4 and ferroptosis-related genes (http://gepia.cancer-pku.cn/) [37].

### 2.8 Cell viability

Following the transfection of cells with negative control siRNA (siNC) and siRNA targeting MCM4 (siMCM4), the cells were seeded into 96-well culture plates at a density of 4 × 10^3^ cells per well. After 24 h, erastin was added to the medium at a concentration of 10.0 μmol/L to induce ferroptosis. The cells were then incubated with erastin for an additional 24 h. Subsequently, 10 μL of CCK-8 solution was added to each well, and the plates were incubated for an additional 2 h. Finally, the absorbance was measured using a microplate reader (Bio Tek) at a wavelength of 450 nm to assess cell viability.

### *2.9* Determination of intracellular reactive oxygen species (ROS)

Intracellular reactive oxygen species (ROS) levels were detected by flow cytometry and fluorescence microscopy using 2′,7′-dichlorofluorescin diacetate (DCFH-DA) as the fluorescent probe. Briefly, cells were incubated with 5 μM DCFH-DA in serum-free medium for 30 min at 37 °C in the dark. After the incubation period, the cells were washed with phosphate-buffered saline (PBS) and collected for quantitative analysis of DCFH-DA fluorescence intensity using a flow cytometer (BD FACSCanto™ II, BD Biosciences, San Jose, CA, USA). Fluorescence microscopy images were also acquired using an EVOS® FL Auto 2 Imaging System (Thermo Fisher Scientific, Waltham, MA, USA) to qualitatively assess intracellular ROS levels based on the DCFH-DA fluorescence signal.

### 2.10 Determination of glutathione GSH

Intracellular glutathione levels were measured using a Glutathione Assay Kit (Beyotime). A375 and B16F0 cells were treated with erastin for 24 h according to the manufacturer's instructions provided with the kit. Briefly, cells were lysed, and the lysates were deproteinized using a reagent provided in the kit. The deproteinized lysates were then incubated with a luminescent substrate that generates a luminescent signal proportional to the amount of glutathione present in the sample. Luminescence was measured using a microplate reader (SpectraMax® iD3 Multi-Mode Microplate Reader, Molecular Devices, San Jose, CA, USA), and glutathione levels were quantified based on a standard curve generated using known concentrations of glutathione provided with the kit.

### 2.11 Lipid peroxidation

Lipid peroxidation was assessed by measuring malondialdehyde (MDA) levels using a Lipid Peroxidation Assay Kit (Beyotime). A375 and B16F0 cells were treated with erastin for 24 h, followed by cell lysis and sample preparation according to the manufacturer's instructions provided with the kit. Briefly, the MDA in the samples reacts with thiobarbituric acid (TBA) to generate a colored product, whose absorbance is measured spectrophotometrically. The samples were incubated with the TBA reagent at 95 °C for a specific duration, and the reaction was terminated by cooling on ice. After centrifugation, the supernatants were transferred to a 96-well plate, and the absorbance was measured at 535 nm using a microplate reader (SpectraMax® iD3 Multi-Mode Microplate Reader). The MDA levels were quantified based on a standard curve generated with known concentrations of MDA provided with the kit.

### 2.12 Western blot assay

Cells were lysed using radioimmunoprecipitation assay (RIPA) lysis buffer (Beyotime), and protein concentrations were determined using a BCA Protein Assay Kit (Solarbio Science & Technology Co., Ltd., Beijing, China). Subsequently, 15 μg of denatured protein samples were loaded onto 10 % SDS-polyacrylamide gels for electrophoretic separation, and the separated proteins were transferred onto 0.45 μm pore size wet polyvinylidene fluoride (PVDF) membranes (Millipore, Burlington, MA, USA). The membranes were subsequently blocked for 1 h at room temperature with QuickBlock™ Blocking Buffer (Beyotime). This was followed by incubation with primary antibodies diluted in blocking buffer for 12 h at 4 °C, including mouse anti-MCM4 (1:1000 dilution, Proteintech, Wuhan, China), anti-GPX4 (1:1000 dilution, Proteintech), and anti-GAPDH (1:5000 dilution, Proteintech) as a loading control. After the membranes were rinsed three times with Tris-buffered saline containing Tween-20 (TBST) buffer, they were incubated with an HRP-conjugated goat anti-mouse IgG secondary antibody (1:5000 dilution, Proteintech) for 1 h at room temperature. Finally, the blots were visualized using an Enhanced Chemiluminescence Detection Kit (Thermo Fisher Scientific), and the protein bands were imaged using a ChemiDoc™ XRS + System (Bio-Rad, Hercules, CA, USA).

### 2.13 In vitro evaluation of antitumor efficiency

#### 2.13.1 Gene delivery efficiency

Gene delivery efficiency was qualitatively analyzed using a pGFP plasmid encoding green fluorescent protein (GFP). Cells were seeded in 24-well plates at a density of 5 × 10^4^ cells per well and incubated for 24 h to allow cell attachment. Then, the cells were incubated with the pGFP plasmid complexed with the respective transfection reagents or nanoparticle formulations according to the desired experimental conditions. After 12 h of incubation, the medium in each well was replaced with fresh DMEM supplemented with 10 % fetal bovine serum (FBS) and 1 % penicillin-streptomycin. The cells were then incubated at 37 °C in a humidified 5 % CO_2_ incubator for an additional 24–48 h to allow GFP expression. Finally, the GFP fluorescence in the transfected cells was visualized and imaged using a fluorescence microscope (Zeiss Axio Observer Z1, Carl Zeiss AG, Oberkochen, Germany) to qualitatively assess the transfection efficiency.

#### 2.13.2 Live and dead cell staining

A375 and B16F0 cells were cultured in 6-well tissue culture plates. Cell viability was assessed by evaluating the integrity of the cell membranes using the Live/Dead® Cell Viability/Cytotoxicity Kit (Yeasen Biotechnology (Shanghai) Co., Ltd., China). After the desired treatments, the cells were incubated with 2 μM Calcein-AM and 4 μM Ethidium homodimer-1 (EthD-1) in phosphate-buffered saline (PBS) for 15 min at room temperature in the dark. The cells were then carefully washed three times with sterile PBS to remove excess dye. Fluorescence microscopy imaging was performed using an EVOS® FL Auto 2 Imaging System (Thermo Fisher Scientific). Live cells were identified by bright green fluorescence emitted by Calcein-AM, which is retained within cells with intact membranes, while dead cells were identified by bright red fluorescence emitted by EthD-1, which penetrates and binds to the nucleic acids of cells with compromised membranes.

### 2.14 In vivo animal experiments

#### 2.14.1 Electrospinning *in vivo* absorption experiment

All animal experiments were conducted in accordance with protocols approved by the Institutional Animal Care and Use Committee of Shantou University Medical College, adhering to ethical guidelines for animal research. For the *in vivo* electrospun implant degradation study, healthy female C57BL/6 mice (18–22 g, 6–8 weeks old) were obtained from Zhejiang Weitong Lihua Experimental Animal Technology Co., Ltd. (Zhejiang, China). Under aseptic conditions, the mice were anesthetized, and the left inguinal region was shaved and disinfected with povidone-iodine solution. A small incision (approximately 0.5 cm) was made in the skin, and the subcutaneous tissue was carefully dissected to create a pocket between the skin and the underlying muscle fascia. The sterilized electrospun material was implanted into the subcutaneous pocket, and the incision was closed in two layers using resorbable sutures for the subcutaneous tissue and skin. The surgical site was then disinfected again with povidone-iodine solution. At predetermined time points (3 and 7 days post-implantation), the mice were euthanized, and the implantation sites were excised for histological analysis and evaluation of the degradation of the electrospun implants.

#### 2.14.2 In vivo antitumor efficacy

For the *in vivo* antitumor study, C57BL/6 female mice were subcutaneously inoculated with B16F0 melanoma cells and randomly divided into five treatment groups (n = 5 per group): NF (nanofiber control), MOF-NF, D@MOF-NF, G@NF, and G-D@MOF-NF. A suspension of B16F0 cells (1 × 10^6^ cells in 100 μL) was injected subcutaneously into the left inguinal region of each mouse. After 3–5 days, when palpable black linear tumors appeared, the mice were anesthetized under aseptic conditions. A small incision (approximately 0.5 cm) was made at the tumor site, and the respective electrospun nanofiber membranes were implanted into the subcutaneous pocket. The incisions were then closed aseptically using resorbable sutures. The mice were monitored daily for their general health and tumor growth. At 28 days post-implantation, the mice were euthanized via CO_2_ asphyxiation. Blood samples were collected from the tail vein, and the tumors and major organs (liver, lungs, spleen, kidneys) were carefully excised. Inflammatory cytokines (IL-1β, IL-6, and TGF-β) were measured by ELISA kits (Zeye Biotech, Shanghai, China). Tumor dimensions were measured using a digital caliper, and a portion of the fresh tumor tissue was snap-frozen at −80 °C for further analyses. The remaining tumor tissues and organs were fixed in 4 % paraformaldehyde (PFA) for subsequent histological analyses.

#### 2.14.3 Effect of MCM4 silencing on ferroptosis induction *in vivo*

For the *in vivo* assessment of the effect of MCM4 silencing on ferroptosis, C57BL/6 female mice were subcutaneously inoculated with B16F0 melanoma cells (1 × 10^6^ cells in 100 μL) and sh-NC (stably expressing control shRNA) or sh-MCM4 (stably expressing MCM4-targeting shRNA). One week post-inoculation, each mouse was administered erastin (50 mg/kg/day) for three weeks. At 28 days post-implantation, the mice were euthanized via CO_2_ asphyxiation. The tumors were carefully excised for subsequent analysis.

### 2.15 Hematoxylin and eosin (H&E) staining

The samples were fixed in 4 % PFA and embedded in paraffin wax. The paraffin-embedded sections were deparaffinized by immersion twice in xylene for 10 min each. Rehydration was performed by sequentially passing the slides through a graded series of ethanol solutions (100 %, 95 %, 85 %, and 75 %) for 3 min per gradient. The slides were then rinsed in distilled water for 2 min. For H&E staining, the sections were stained with hematoxylin solution for 10 min and washed under running distilled water to remove excess stain. Differentiation was achieved by dipping the slides in acid‒alcohol solution (60 drops) followed by washing under running tap water or distilled water for 5 min twice. The sections were then stained with eosin dye solution for 120 s, and the excess dye was removed. Dehydration was performed by sequentially passing the slides through a graded series of ethanol solutions in the reverse order (75 %, 85 %, 95 %, and 100 %) for 5 min per gradient. The sections were cleared by immersion in xylene twice for 1 min each. Finally, the slides were mounted with a neutral mounting medium, and the stained sections were observed under a light microscope.

### 2.16 Statistical analysis

The results are presented as the means ± standard deviations (SD). Statistical analysis was performed using a two-tailed Student's *t*-test for comparisons between two groups or one-way analysis of variance (ANOVA) followed by Tukey's post hoc test for multiple group comparisons. Differences were considered statistically significant at p < 0.05 (*), p < 0.01 (**), and p < 0.001 (***). All the statistical analyses were carried out using GraphPad Prism software (version 9.0, GraphPad Software, San Diego, CA, USA).

## 3 Results

### 3.1 Co-expression of MCM4 and ferroptosis-related genes

Ferroptosis is emerging as a promising therapeutic approach for cancer treatment [38]. Several anti-cancer genes and oncogenic signals modulate ferroptosis, with cancer cells showing heightened susceptibility due to their distinct metabolic characteristics and specific genetic alterations [39]. Notably, drug-resistant tumor cells and those undergoing epithelial–mesenchymal transition, often associated with increased metastatic potential, are more sensitive to ferroptosis induction [40]. Oncogenic mutations reprogramme the metabolic pathways of tumor cells to meet their elevated nutritional and energy demands, often increasing their vulnerability to ferroptosis [41]. For instance, undifferentiated melanoma subtypes are characterized by an accumulation of polyunsaturated fatty acids and a deficiency in reduced GSH, increasing susceptibility to ferroptosis [42]. Consequently, we investigated the relationship between MCM4 and ferroptosis. Analysis of the mRNA expression levels of MCM4 in MM and normal tissues in the GEPIA database revealed significantly upregulated MCM4 expression in MM tissues compared to normal tissues (Fig. S2). Furthermore, GEPIA revealed co-expression relationships between MCM4 and key ferroptosis-related genes in melanoma patients, including positive correlations with ACSL4 [43], TP53, TFRC, GPX4, FTH1 [44], BCL-2, and SLC7A11 [45] and a negative correlation with LPCAT3 (Fig. S2), indicating the positive association of MCM4 with ferroptosis-regulating genes.

**Fig. 2:**
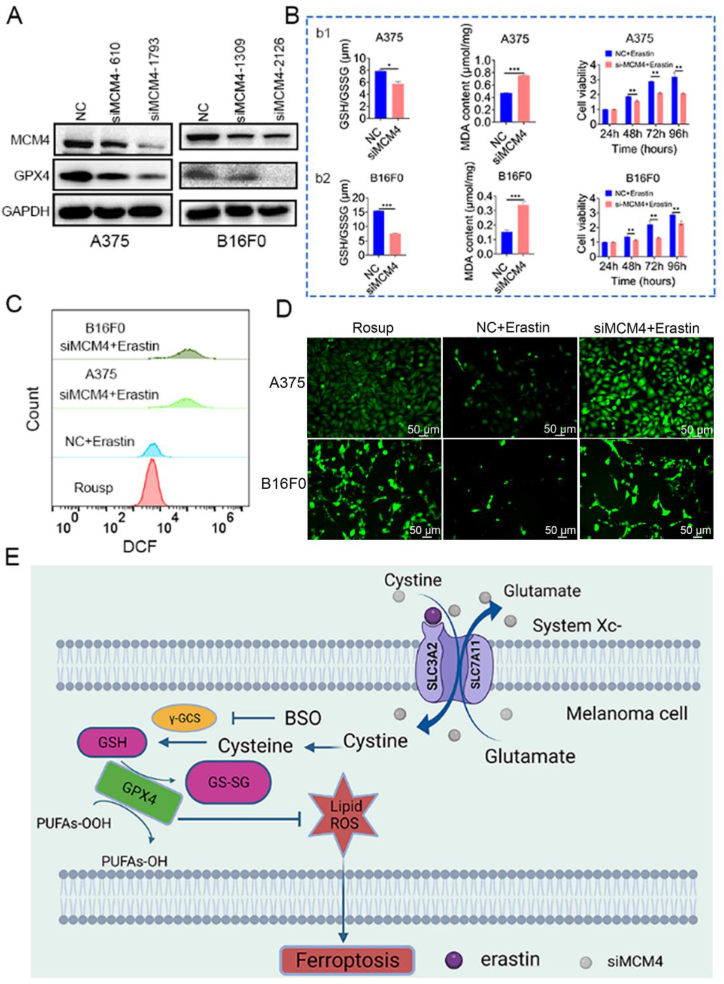
*MCM4 knockdown sensitizes melanoma cells to erastin-induced ferroptosis.* (A) Western blot results showing the expression of GPX4 after the knockdown of MCM4 in A375 and B16F0 cells. (B) The reduction in GSH and MDA levels and melanoma cell viability after knockdown of the MCM4 gene. (C) Flow cytometry analysis of cellular ROS levels in A375 and B16F0 cells after MCM4 silencing and erastin treatment for 24 h. Rosup was used as a positive control. (D) Fluorescence microscopy images of cellular ROS in A375 and B16F0 cells after MCM4 silencing and erastin treatment for 24 h. (E) Schematic illustration of the underlying signaling pathway involved in the interaction between MCM4 and ferroptosis.

Next, we delivered si-NC or si-MCM4 encapsulated with a plasmid encoding GFP to A375 cells. Fluorescence images confirmed the high transfection efficiency (Fig. S3). Western blot results showed that silencing MCM4 led to reduced GPX4 expression in both human and murine melanoma cells (Fig. 2A). si-MCM4-1793 and si-MCM4-2126, which had high knockdown efficiency, were used for the following experiments. Ferroptosis is regulated by multiple pathways, including the GPX4-regulated pathway, iron metabolism pathway, and lipid metabolism pathway [46]. GPX4 serves as a primary defense protein against ferroptosis and is an antioxidant enzyme that directly neutralizes lipid peroxides [47]. The above data suggest that MCM4 is a positive regulator of GPX4 expression in melanoma cells.

### 3.2 MCM4 knockdown sensitizes melanoma cells to erastin-induced ferroptosis

Ferroptosis, a distinct form of regulated cell death, predominantly occurs due to the excessive accumulation of ROS and lipid peroxidation [48]. GSH, an essential intracellular antioxidant, plays a pivotal role in eliminating lipid peroxides through its involvement in GPX4 activity [49]. Our results demonstrated a significant decrease in GSH levels after MCM4 silencing. In contrast, the level of the lipid peroxidation byproduct MDA was significantly increased after MCM4 knockdown (Fig. 2B). Furthermore, the CCK-8 assay results showed a significant decrease in cell viability in the si-MCM4 group when the cells were exposed to erastin (Fig. 2B). These findings suggest that MCM4 knockdown increases the sensitivity of melanoma cells to the ferroptosis-inducing effects of erastin, indicating that MCM4 plays a protective role against ferroptosis in melanoma cells.

We further detected changes in ROS levels by the DCFDA probe in erastin-treated melanoma cells with or without MCM4 knockdown. The results demonstrated a marked increase in intracellular ROS levels in the A375 and B16F0 melanoma cell lines upon MCM4 depletion and subsequent treatment with the ferroptosis inducer erastin (Fig. 2C and D). In summary, our findings revealed a strong association between MCM4 expression and susceptibility to ferroptosis in melanoma cells. Fig. 2E illustrates the proposed underlying signaling pathway through which MCM4 modulates ferroptosis by targeting GPX4 in melanoma cells.

### 3.3 In vitro evaluation of co-delivery of gene and chemotherapy drugs

**Fig. 3:**
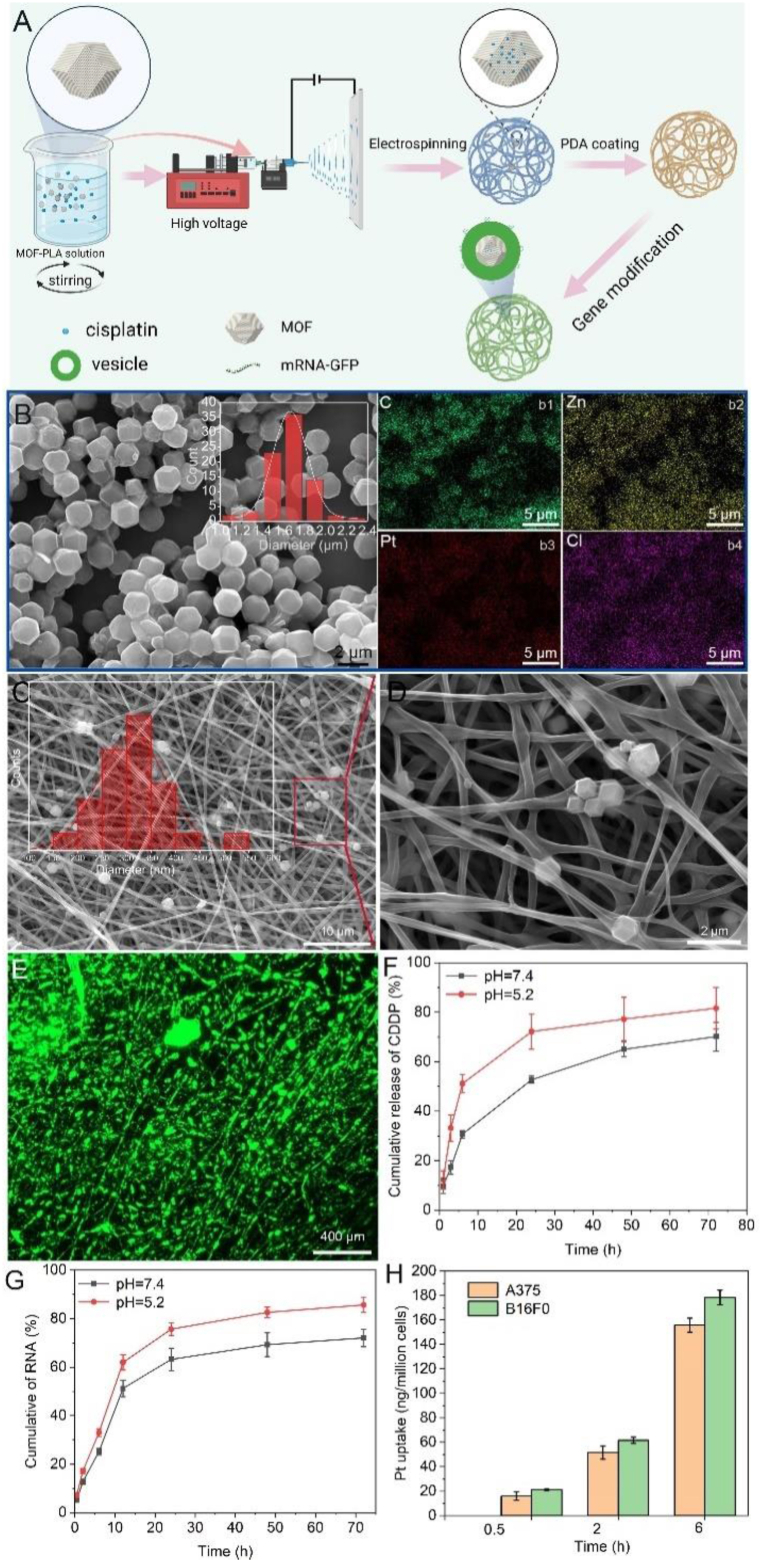
Sample preparation and characterization. (A) Schematic diagram of the design and synthesis of the G-D@MOF-NF membrane. (B) SEM image and corresponding EDX spectrum of drugs loaded within MOF particles (inset graph: diameter distribution of MOFs). (C) and (D) SEM images of the G-D@MOF-NF membrane. (E) Fluorescence image of FAM-labeled RNA on the G-D@MOF-NF membranes. (F) Cisplatin and (G) mRNA Release test in both pH 7.4 and pH 5.2 (H) Platinum uptake test in B16F0 and A375 cells.

For effective treatment and prevention of recurrence at residual tumor sites, drug delivery systems should integrate both short-term and continuous treatment strategies to achieve both rapid *in situ* eradication of tumor tissue and long-term tumor suppression. In this regard, PLA, a biodegradable and non-toxic material approved by the Food and Drug Administration (FDA), was chosen for electrospinning fiber membrane fabrication [50,51]. Electrostatic spinning technology is employed to fabricate a 3D structured membrane. MOFs are characterized by high porosity, substantial drug-loading capacity, efficient encapsulation of small and large biomolecules, pH-responsive degradation, and drug release properties [52]. As shown in Fig. 3A, PLA and a solution of drug-loaded zeolitic imidazolate framework-8 (ZIF-8) were mixed by ultrasonic dispersion and subsequently spun by electrospinning. Furthermore, the spun membrane was modified with polydopamine (PDA) and further functionalized with liposome-encapsulated messenger RNA (mRNA). SEM analysis revealed that the MOF particles were micron-sized and monodisperse with a diameter of 1.7 ± 0.1 μm. Energy-dispersive X-ray spectroscopy (EDX) confirmed the uniform distribution of C, Zn, Pt, and Cl within the MOFs, indicating effective adsorption of the drug onto the MOF particles (Fig. 3B). Moreover, MOF particles were evenly distributed within the PLA membrane (Fig. 3C), and higher magnification images showed that the MOFs were encapsulated within the fibers (Fig. 3D). Fluorescence image demonstrated that the mRNA was successfully decorated on the G-D@MOF-NF membrane (Fig. 3E). According to the UV spectrum of cisplatin, it can be seen that it has an obvious ultraviolet absorption peak at 252 nm (Fig. S4). Moreover, the cisplatin and mRNA could sustainably release from the fibrous membranes for more than 72 h. Compared to a neutral environment, mRNA and drugs are released more quickly under weakly acidic conditions, nearly 75 % release within 24 h in acid condition (Fig. 3F, G). The results showed that mRNA and cisplatin could be effectively released from the spinning membrane and rapidly released in a weakly acidic environment that mimics tumors, and played a role in the postoperative treatment of tumors. We further tested the phagocytosis of the cells against cisplatin. As the culture time increases, more and more cisplatin was uptake by the B16F0 and A375 cells, and nearly 160 ng of cisplatin can be phagocytosed per million cells after 6 h of incubation (Fig. 3H).

**Fig. 4:**
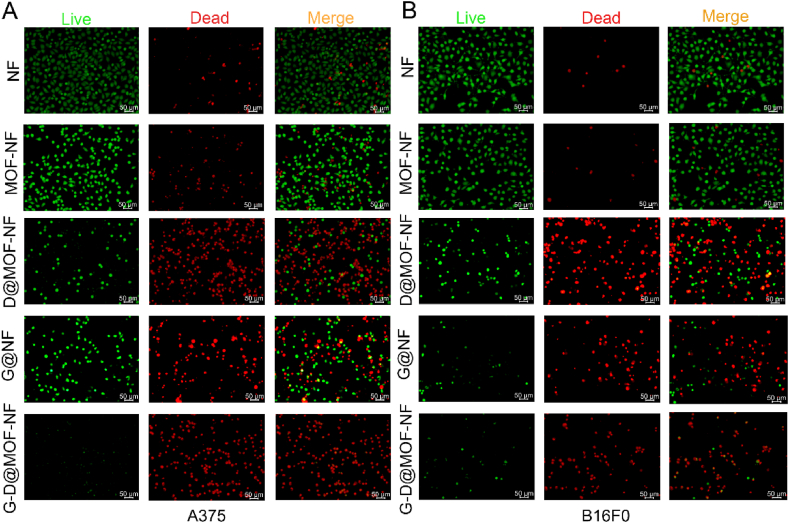
*In vitro* anti-cancer activity of different nanocarriers. Fluorescence images of live/dead staining of (A) A375 and (B) B16F0 cells growing on blank PLA membrane (NF), PLA membrane encapsulating MOF (MOF-NF), cisplatin-loaded MOF (D@MOF-NF), MCM4 siRNA-loaded membrane (G@NF), and cisplatin and siRNA loaded MOF-NF (G-D@MOF-NF). Live cells were stained green, and dead cells were stained red.

Live and dead cell staining demonstrated the therapeutic efficacy of the co-delivered system in melanoma cells. Both A375 and B16F0 cells exhibited high levels of viability on the surface of the PLA membranes (NF group) and MOF-loaded nanofiber membranes (MOF-NF group). Electrospinning membranes with cisplatin (D@MOF-NF) or siMCM4 (G@NF) displayed strong anti-tumor activity, accompanied by a significant amount of cell death. Compared to the other groups, cells growing on the membrane with siMCM4 and drug-loaded MOFs (G-D@MOF-NF group) exhibited the greatest number of dead cells. These findings imply that siMCM4 delivery via the spun membrane enhances the tumoricidal effect of cisplatin on melanoma cells (Fig. 4).

### 3.4 MOF-based co-delivery of cisplatin and siMCM4 has a strong anti-tumor effect *in vivo*

**Fig. 5:**
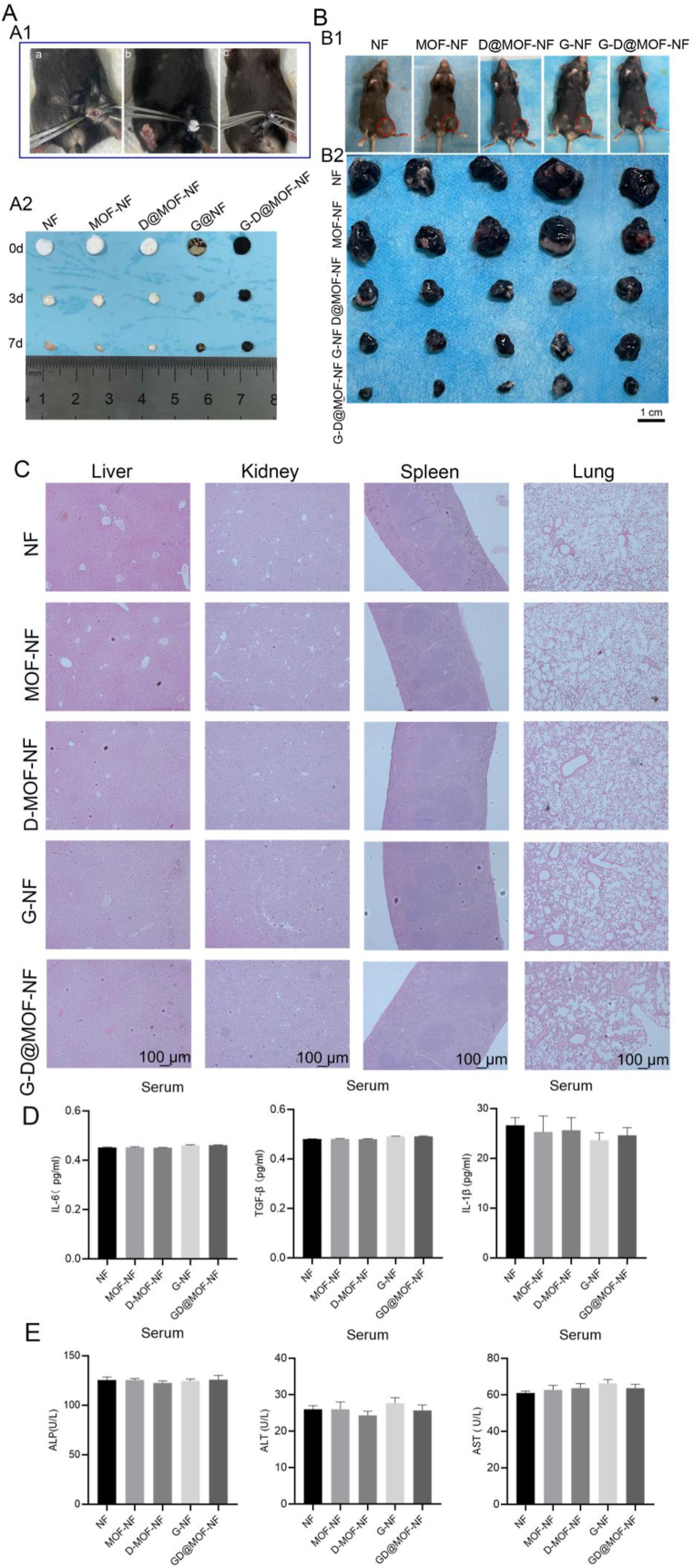
*In vivo* evaluation of anti-cancer effects and bio-safety of the co-delivery strategy. (A) The absorption and degradation effects of samples from mice. (B) Antitumor efficacy evaluation of each group of mice inoculated with B16-F0 melanoma cells. (C) H&E staining of different organs after the administration of different nanocarriers. (D) Detection of inflammatory cytokines (IL-6, TGF-β, and IL-1β) in blood plasma. (E) Analysis of hepatic damage indicators (ALP: Alkaline Phosphatase; ALT: Alanine Aminotransferase; AST: Aspartate Aminotransferase) in the blood plasma.

To assess the *in vivo* degradation kinetics of the spun membranes, the membranes were subcutaneously implanted in mice (as illustrated in Fig. 5A1). The membranes were retrieved on days 3 and 7, and the membrane was reduced to approximately half of its original diameter by day 3 post-implantation, and further degradation was evident by day 7 (Fig. 5A2). This indicates that the PLA-based membrane exhibits favorable *in vivo* degradation, which may lead to the gradual release of therapeutic drugs and genes at the site of implantation.

To evaluate the anti-tumor effect of electrospun carriers on melanoma, B16F0 melanoma cells were inoculated into C57BL/6 mice to establish tumor growth models. The electrospun membranes with or without cisplatin and siMCM4 were implanted at the tumor site. Animals implanted with the electrospun membrane showed no signs of infection or immune rejection. Mice implanted with blank PLA nanofiber membrane (PLA-NF) or MOF-loaded nanofiber membrane (MOF-NF) exhibited significant tumor growth, while mice implanted with cisplatin-loaded MOF (D@MOF-NF) or MCM4 siRNA-loaded membrane group (G@NF) showed a noticeable reduction in tumor size. MOF-NF loaded with cisplatin and siMCM4 (G-D@MOF-NF) exhibited the strongest anti-tumor effect (Fig. 5B). To further evaluate the biosafety of the delivery system, we examined histological changes in major organs (kidney, liver, spleen, and lung) by Hematoxylin and Eosin (H&E) staining. The data showed no abnormal pathological signs in these organs (Fig. 5C). Furthermore, there were no detectable increases in the plasma levels of inflammatory cytokines (IL-6, TGF-β, and IL-1β) (Fig. 5D) or hepatic damage indicators (ALP, ALT, and AST) (Fig. 5E). Together, these data demonstrated the anti-tumor efficacy and biosafety of the G-D@MOF-NF system.

### 3.5 Silencing MCM4 enhances the sensitivity of melanoma to ferroptosis induction *in vivo*

**Fig. 6:**
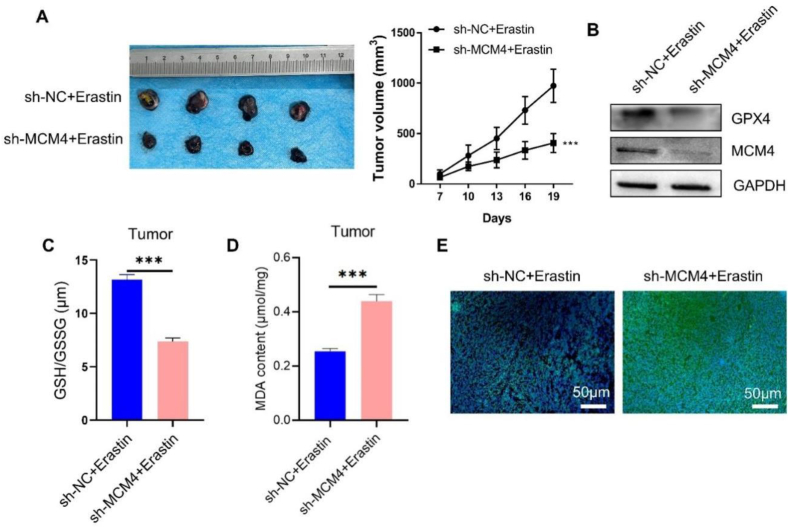
Silencing MCM4 enhances the sensitivity of melanoma to ferroptosis induction *in vivo*. C57BL/6 mice were inoculated with B16F0 melanoma cells transfected with sh-NC or sh-MCM4. One week pos-tinoculation, each mouse was administered erastin for three weeks. (A) Tumor tissue images of the sh-NC and sh-MCM4 groups. (B) Western blot analysis of GPX4 and MCM4 in tumor tissues. (C) Detection of GSH levels in tumor samples. (D) Analysis of MDA levels in tumor tissues. (E) Detection of ROS levels in tumor samples.

To further demonstrate the effect of MCM4 knockdown on erastin-induced cell death *in vivo*, B16F0 melanoma cells were subcutaneously inoculated into C57BL/6 mice with sh-NC (B16F0 melanoma cells stably expressing control shRNA) or sh-MCM4 (B16F0 melanoma cells stably expressing MCM4-targeting shRNA). One week postinoculation, each mouse was administered erastin for three weeks. The tumor size was significantly reduced in the MCM4-silenced group (Fig. 6A). We further observed a decrease in GPX4 levels in tumor samples with MCM4 knockdown (Fig. 6B). Moreover, there was a significant reduction in GSH levels in MCM4-silenced tumor samples (Fig. 6C), while ROS and MDA levels were elevated after MCM4 knockdown (Fig. 6D, E). These data suggest that targeting MCM4 could augment ferroptosis induction by erastin in melanoma.

## 4 Discussion

Traditional cytotoxic drugs and targeted therapies inhibit tumor growth by inducing cancer cell apoptosis [53]. However, the resistance of tumor cells to targeted therapies remains a significant challenge. Exploring non-apoptotic programmed cell death pathways, such as ferroptosis pathways, offers an alternative therapeutic strategy to curb tumor growth [54]. The current study identified MCM4 as a potential target for melanoma therapy, as reducing MCM4 expression enhanced ferroptosis sensitivity in melanoma cells. Furthermore, we demonstrated that the co-delivery of siMCM4 and cisplatin exerts a strong tumoricidal effect in a mouse model of melanoma. Previous studies have supported the efficacy of combined drug delivery systems. Lei et al. found that co-loading polyethyleneimine (PEI)/DNA nanoparticles and the drug viloxazine on poly(lactic-co-glycolic acid) (PLGA) fibers significantly improved the therapeutic efficacy for malignant brain tumors [55]. Achille et al. reported the successful use of biodegradable nanofiber scaffolds for the encapsulation and delivery of biologically active DNA, achieving gene therapy for tumors through effective RNA interference [56]. Yu et al. designed a biomimetic scaffold incorporating copper-doped silica microspheres for synergistic photothermal therapy for melanoma and skin tissue repair [57]. These studies highlight the synergistic effects of combining cytotoxic drugs with gene targeting, underscoring the potential of the co-delivery strategy of siMCM4 and chemotherapeutic drugs to enhance the anti-tumor effect on melanoma.

This study proposed a new therapeutic strategy for diverse drug delivery and spatiotemporal release to prevent post-operative tumor recurrence. We elucidated the potential of targeting MCM4 for melanoma treatment and demonstrated the anti-tumor efficacy both *in vitro* and *in vivo*. Uniform MOF particles can be electrospun into a PLA matrix, maintaining the morphology of drug-loaded MOFs seamlessly integrated into the membrane (Fig. 3). This delivery system enables the simultaneous administration of multiple treatment modalities, including siRNAs, chemotherapeutic agents, ferroptosis inducers, or DNA vaccines. Furthermore, *in vivo* analysis confirmed the biodegradability and safety of the electrospinning membrane drug delivery system. Compared to direct drug administration via intravenous injection or localized injection at the tumor site, loading drugs onto MOFs with a large surface area allows for high-dosage delivery [58]. Furthermore, encapsulating MOFs within the electrospinning membrane ensures the slow and sustained release of the drugs.

While the results of this study are promising, several limitations should be acknowledged. Further studies are needed to validate the efficacy and safety of this co-delivery system in more advanced animal models before considering potential clinical translation. The complexity of the tumor microenvironment and patient-specific factors may influence the therapeutic response, which cannot be fully recapitulated in these preclinical models. The long-term stability and storage conditions of electrospun nanofiber membranes need thorough evaluation, as they could impact shelf life and clinical applicability. Moreover, the specific mechanisms underlying the synergistic anti-tumor effects of cisplatin and MCM4 silencing require further mechanistic investigation. Finally, while the co-delivery system demonstrated biocompatibility in this study, the incorporation of a targeted release strategy, such as pH-dependent release in the tumor microenvironment, may further enhance anti-tumor efficacy and reduce toxicity to normal tissues [59].

## 5 Conclusion

In this study, we reported that MCM4 is a promising target for melanoma-specific therapy, given its crucial role in regulating ferroptosis sensitivity in melanoma cells. We also engineered electrospun nanofibers to integrate drug- and gene-targeting payloads to enhance therapeutic efficacy. The spun membrane exhibited favorable adsorption and storage properties, facilitating the stable, prolonged, and controlled release of chemotherapeutic drugs. Moreover, the nanoparticles encapsulated within the composite fibrous structure through electrospinning demonstrated robust stability and strong anti-cancer effects, both *in vitro* and *in vivo*. While further research is warranted to comprehensively assess the therapeutic potential of this approach in melanoma, the developed material shows outstanding anti-tumor efficacy and favorable biocompatibility *in vivo*. These promising findings underscore the immense potential of leveraging the synergistic co-delivery of siRNA and chemotherapeutics for cancer treatment.

## Data availability

The single‐gene bioinformatics datasets analyzed in the present study are available from GEPIA (http://gepia.cancer-pku.cn/).

## Ethics approval

All animal handling and procedures were approved by the Animal Care and Use Committee of the Medical College of Shantou University (Number:SUMC2023-419).

## CRediT authorship contribution statement

**Xuewei Zhang:** Writing – original draft, Methodology, Formal analysis, Data curation, Conceptualization. **Guoxing Zheng:** Methodology. **Zibin Zhou:** Methodology. **Mingyu Zhu:** Writing – review & editing, Conceptualization. **Shijie Tang:** Writing – review & editing, Conceptualization.

## Declaration of competing interest

The authors declare that they have no known competing financial interests or personal relationships that could have appeared to influence the work reported in this paper.
