# Supplementary material for: Co-delivery of siRNA and cisplatin via electrospun Nanofibrous membranes for synergistic treatment of malignant melanoma
Source: Heliyon. 2024 Sep 6;10(17):e37517. doi: 10.1016/j.heliyon.2024.e37517 (PMC11407083; doi:10.1016/j.heliyon.2024.e37517)
Supplement: Multimedia component 1 [file mmc1.docx]

**Co-Delivery of siRNA and Cisplatin via Electrospun Nanofibrous Membranes for Synergistic Treatment of Malignant Melanoma**

Xuewei Zhang^a^, Guoxing Zheng^b^, Zibin Zhou^b^, Mingyu Zhu^c,d*^, Shijie Tang^a*^

^a^ Department of Plastic Surgery and Burn Center, Second Affiliated Hospital, Shantou University Medical College, Shantou, Guangdong, 515000, China;

^b^ Department of Spine Surgery, Second Affiliated Hospital, Shantou University Medical College, Shantou, Guangdong, 515000, China;

^c^ Division of Pharmaceutical Chemistry and Technology, Faculty of Pharmacy, University of Helsinki, Helsinki 00014, Finland;

^d^ Department of Materials Science and Engineering, Southern University of Science and Technology, Shenzhen, Guangdong 518055, China


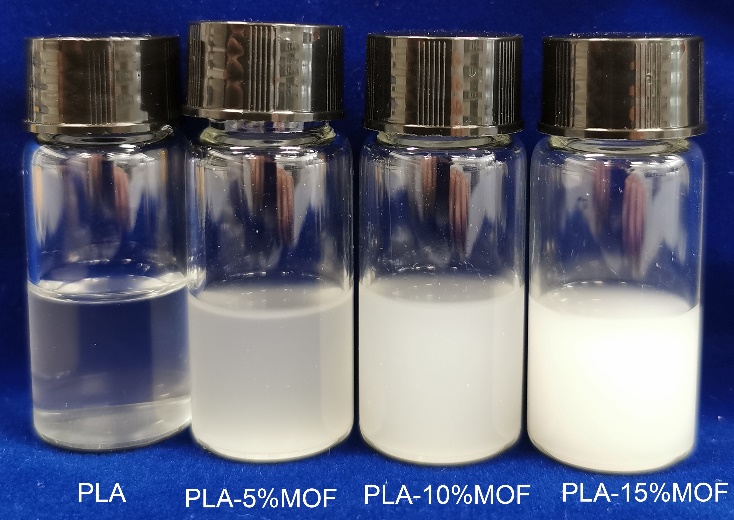


**Fig.S1** The photos of different MOF content in PLA solution.

**Table S1 The target sequence of short hairpin RNA MCM4:**

| Gene name | sense | antisense |
| --- | --- | --- |
| MCM4-Mus-1309 | GCAGACACCUCACACUAUUTT | AAUAGUGUGAGGUGUCUGCTT |
| MCM4-Mus-2126 | CCGCACACAUUGUUGUCAATT | UUGACAACAAUGUGUGCGGTT |
| MCM4-Homo-610 | GCAGAAGAUAUAGUGGCAATT | UUGCCACUAUAUCUUCUGCTT |
| MCM4-Homo-1793 | GCCAGUACACGUCUGGGAATT | UUCCCAGACGUGUACUGGCTT |


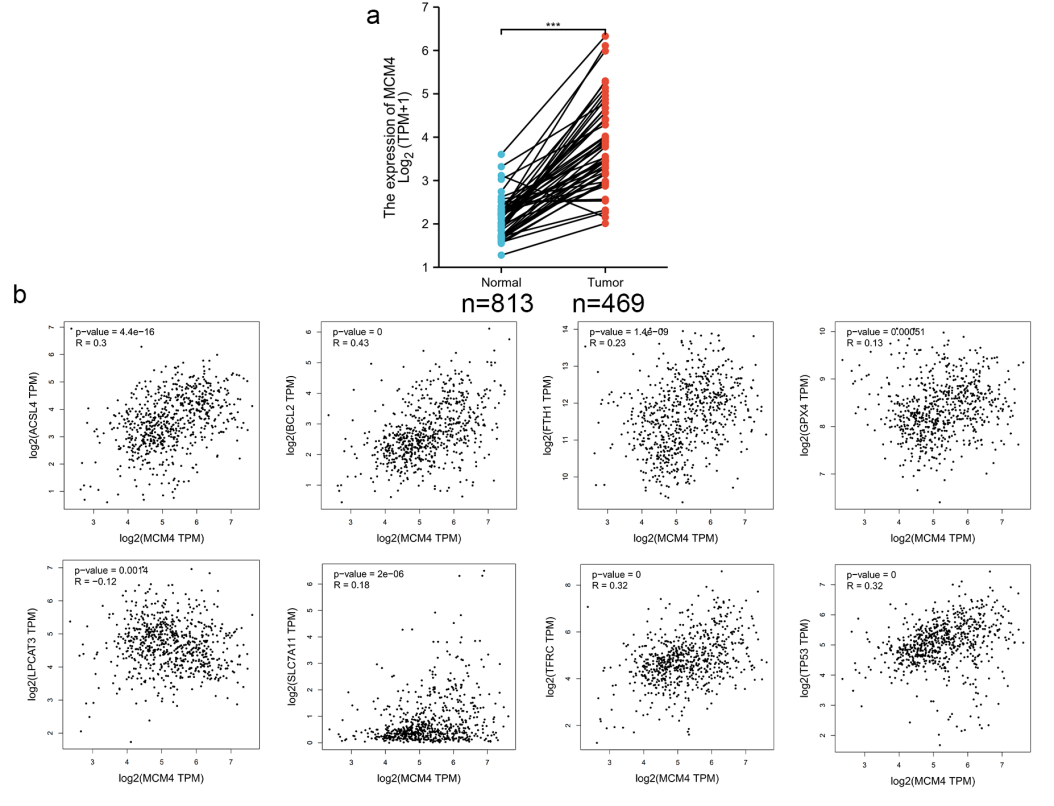


**Fig. S2** The expression of MCM4 mRNA in MM (a) and the co-expression of genes related to ferroptosis were analyzed by the GEPIA database (b).


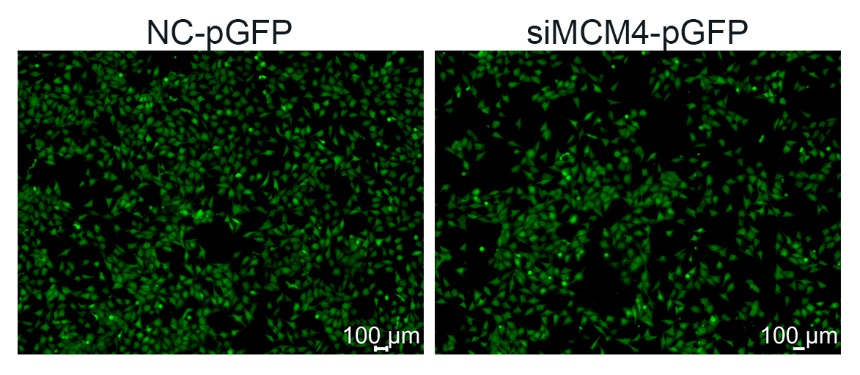


**Fig. S3** Fluorescence microscopy image of melanoma cells transfected.

**
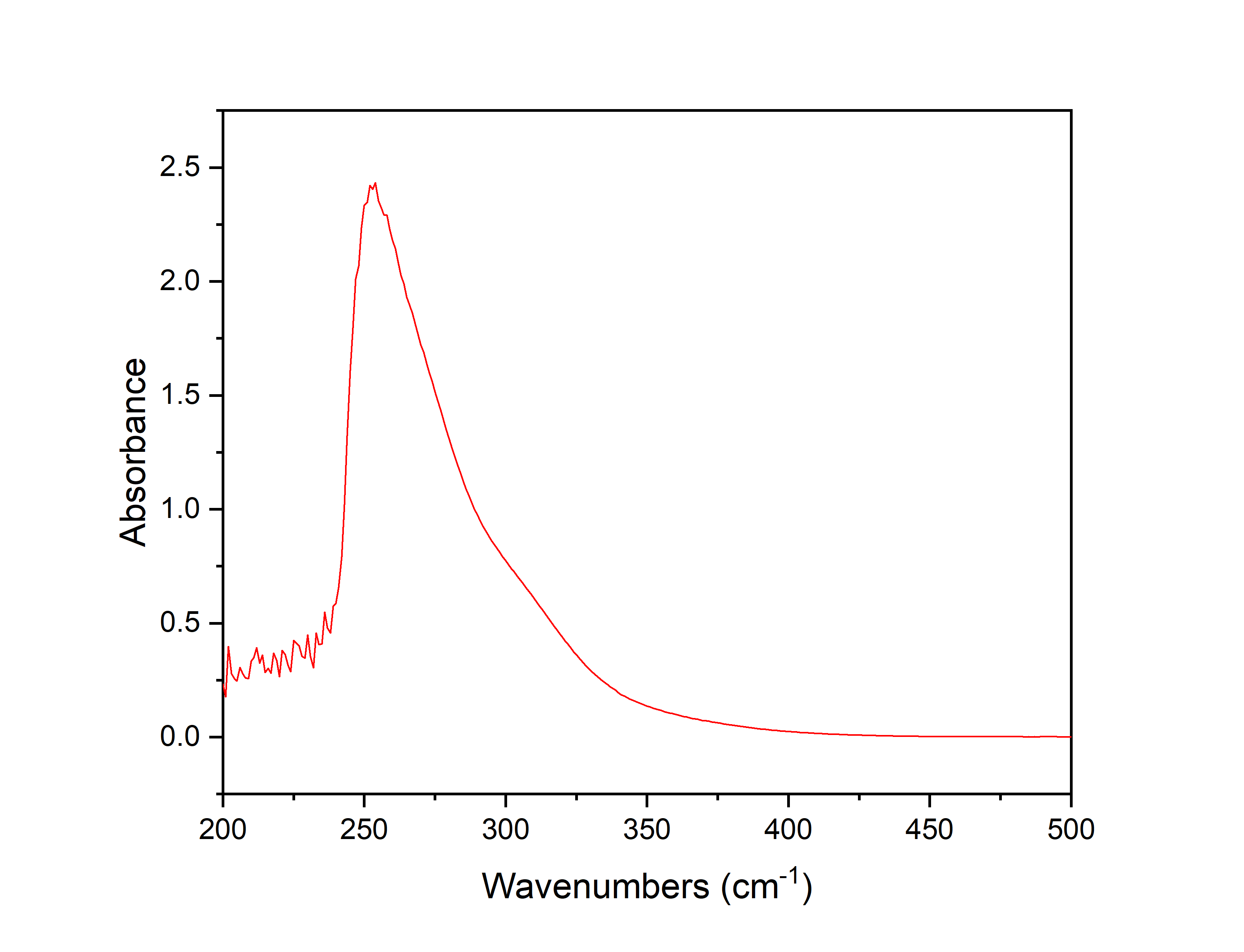
**

**Fig. S4** The UV spectrum of cisplatin (CDDP).
